# Improving Oxygen Permeability and Thermostability of Polycarbonate via Copolymerization Modification with Bio-Phenol Polysiloxane

**DOI:** 10.3390/polym11081302

**Published:** 2019-08-03

**Authors:** Xiaoyan Pang, Xin Ge, Jianye Ji, Weijie Liang, Ruoling Liu, Xunjun Chen, Guoqiang Yin, Jianfang Ge

**Affiliations:** 1 College of Chemistry and Chemical Engineering, Zhongkai University of Agriculture and Engineering, Guangzhou 510230, China; 2 School of Materials Science and Engineering, Northwestern Polytechnical University, Xi’an 710072, China; 3 Guangzhou Key Laboratory for Efficient Utilization of Agricultural Chemicals, Guangzhou 510225, China

**Keywords:** bio-phenol polysiloxane, polycarbonate, copolymer, structure, thermostability

## Abstract

As a new kind of functionalized polysiloxane with chemical reactivity, bio-phenol polysiloxane was synthesized through facile heterogeneous catalytic route. Bio-phenol polysiloxane/polycarbonate (Si/PC) block copolymer was synthesized via a three-step approach, and the effect of the amount of bio-phenol polysiloxane on the properties of Si/PC copolymer was then studied. The structure and morphology of Si/PC copolymer were characterized, showing that, when the amount of bio-phenol polysiloxane reached 20%, the pyrolysis temperature of Si/PC copolymer at 5% weight loss was 450.8 °C which was 76.1 °C higher than pure PC. The oxygen permeability of 20%Si/PC copolymer membrane was 502.65 cm^3^/m^2^·24h·0.1MPa, which was increased by 128.4% compared with pure PC membrane. The mechanical property and hydrophobicity of Si/PC copolymer had been improved.

## 1. Introduction

Derived from natural products, eugenol (4-allyl-2-methoxyphenol) is one kind of bio-phenol, which is a primary constituent of plant essential oil, such as clove oil, laurel oil, and camphor oil and characterized by the presence of functional groups, such as the hydroxy, methoxy and allyl groups. Eugenol has been used in compound modification for its antibacterial activity, thermal stability, and chemical reactivity [1,2,3,4,5]. With the reactive phenol hydroxyl, bio-phenol polysiloxane has been synthesized via facile heterogeneous catalytic route between eugenol and hydrogen terminated polysiloxane [6,7,8]. As a new kind of functionalized polysiloxane, bio-phenol polysiloxane, which has chemical reactivity and antibacterial activity, has been used in the polymer modification [9]. Chen [10] used eugenol-modified polysiloxanes as effective anticorrosion additives for epoxy resin coatings, and the result showed that the eugenol-modified polysiloxane additives can significantly increase the performance of epoxy resin coatings in terms of thermal stability, hydrophobicity, and resistance to water penetration. Xu [11] synthesized poly(aryl ether sulfone)s incorporating cage and liner organsiloxane in the backbones, and the result showed the copolymer had a comprehensive property.

Polycarbonate (PC) is an engineering plastic which exhibits outstanding impact strength and dimensional stability [12,13,14]. However, having high melt viscosity, PC is difficult to process and easy to stress crack [15]. The phenolic hydroxyl group of bio-phenol polysiloxane could copolymerize with the PC monomers via the transesterification method to synthesize bio-phenol polysiloxane/polycarbonate (Si/PC) copolymer and thus improve the properties of PC to meet the use requirements in cutting-edge technology [16,17]. Kopylov [18] synthesized new linear polycarbonate-polysiloxanes based on oligomeric organosilicon bis phenols and has studied the molecular weight and mechanical characteristic of copolymers.

In this paper, bio-phenol polysiloxane was synthesized through the facile heterogeneous catalytic route and designed to copolymerize with the PC monomers via a three-step approach to synthesize the Si/PC copolymer. The obtained Si/PC copolymer was characterized by Fourier transform infrared spectrometer (FTIR), nuclear magnetic resonance hydrogen spectrometer (^1^HNMR), gel permeation chromatography (GPC), transmission electron microscopy (TEM) and scanning electron microscopy (SEM). The thermal stability, mechanical properties, oxygen permeability, and hydrophobicity of the Si/PC copolymer were also measured.

## 2. Materials and Methods

### 2.1. Materials

Octamethylcyclotetrasiloxane (D_4_) and 1,1,3,3-tetramethyldipolysiloxane (HMMH) were obtained from Shenzhen Ji-Peng Silicon Fluoride Materials Corporation (Shenzhen, China). The macroporous cationic resin was gained from Jiangyin Nanda Synthesis Chemical Corporation (Jiangyin, China). Eugenol of 98% purity was purchased from Guangdong Tongcai New Material Corporation (Guangzhou, China). Carbon nanotube supported platinum catalyst (Pt-CNT) with 3% Pt was prepared in Zhongkai University of Agriculture and Engineering (Guangzhou, China) [6]. Diphenyl carbonate of 99% purity was obtained from Guangdong Wengjiang Reagent Corporation (Shaoguan, China). Lithium acetate was gained from Shanghai Eppie Chemical Reagent Corporation (Shanghai, China) and bisphenol A was purchased from Xi’an Tianmao Chemical Corporation (Xi’an, China).

### 2.2. Synthesis of Bio-phenol Polysiloxane/Polycarbonate Block Copolymer

D_4_ (122.1 g, 0.4125 mol), HMMH (4.02 g, 0.03 mol), and macroporous cationic resin (3.78 g, 3 wt %) were added into a 250 mL three-necked flask. The reaction was carried out at 80 °C for 4 h and then the obtained product purified. Finally, the clear liquid was hydrogen terminated polysiloxane.

Hydrogen terminated polysiloxane (80 g, 0.02 mol) and eugenol (6.56 g, 0.04 mol) were placed in a 150 mL three-necked flask. After venting nitrogen for 30 min, the catalyst Pt-CNT was added into the flask. And then the reaction system was heated to 80 °C under nitrogen atmosphere and reacted for 6 h. Bio-phenol polysiloxane was obtained after centrifugation for 1 h.

**Figure 1:**
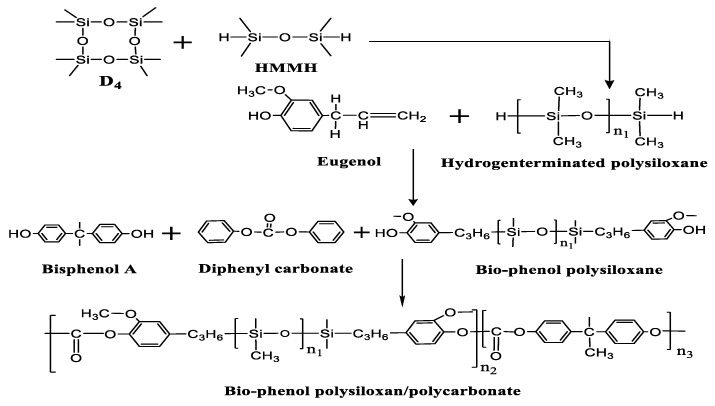
The synthetic equation of bio-phenol polysiloxane/polycarbonate block copolymer.

Diphenyl carbonate (39 g), bisphenol A (39.86 g), lithium acetate (0.005 g), and bio-phenol polysiloxane were added into the three-necked flask. After venting nitrogen for 30 min, the system was heated to 200 °C and reacted for 3 h. The obtained copolymer was dissolved in the methylene chloride, and the insoluble substances were removed by filtration. The filtered filtrate was washed into cyclohexane, and a white precipitate was obtained by filtration. The obtained white precipitate was dissolved again in methylene chloride and washed with cyclohexane three times, and then placed into the drying cabinet to dry for 12 h. By adjusting the content of bio-phenol polysiloxane, 0%Si/PC, 10%Si/PC, 14%Si/PC, 18%Si/PC, 20%Si/PC copolymer were synthesized, and the percentages represented the content of the bio-phenol polysiloxane. Figure 1 shows the synthesis of the bio-phenol polysiloxane/polycarbonate block copolymer.

### 2.3. ^1^H NMR Analysis

The ^1^H NMR spectra were recorded on a Bruker Advance Ⅲ HD 500 spectrometer (Bruker Corporation, Karlsruhe, Germany) using CDCl_3_ as a solvent. The chemical shifts relative to tetramethylsilane, which is used as an internal reference.

### 2.4. FTIR Analysis

FTIR on the Si/PC copolymer was performed by using an FTIR spectrum 100 (Perkin-Elmer Corporation, Fremont, CA, USA) at room temperature. Before scanning, the samples were dried in the drying cabinet at 80 °C for 4 h to remove moisture. The samples were scanned within the range from 400 to 4000 cm^−1^.

### 2.5. GPC Analysis

Gel permeation chromatograms were obtained using a gel permeation chromatograph (Waters 1515 GPC, Waters, Milford, MA, USA) with tetrahydrofuran as an eluent, at a flow rate of 1 mL·min^−1^ using polystyrene as a standard.

### 2.6. SEM Characterization

The cross-sectional of Si/PC copolymer membrane was prepared by freezing in liquid nitrogen and was observed by scanning electron microscopy (EVO 18, Carl Zeiss, Jena, Germany) at a 15-kV accelerating voltage. Before the SEM observations, the samples were coated with a fine gold layer for 45 s.

### 2.7. TEM Characterization

TEM images were taken on a transmission electron microscope (JEM 100CX, Leica, Germany). The samples were prepared by solution method and dissolved in tetrahydrofuran. 3–4 drip of the solution was dropped on the copper net and then dried for 30 min before observing.

### 2.8. Thermal Analysis

Thermogravimetric analysis was carried out using a Mettler Toledo TG/DTA thermal analyzer (Mettler-Toledo AG Corporation, Columbus, OH, USA) to measure the temperature of the Si/PC copolymer from the beginning of weight loss to 100% weight loss. The experiments were performed in the range of 40 to 900 °C at a heating rate of 10 °C/min under nitrogen atmosphere.

### 2.9. Measurement of Mechanical Property

The mechanical properties of Si/PC copolymer membranes were tested on a microcomputer-controlled electronic universal testing machine (CMT6503, Shenzhen MTS Test Machine Company Ltd., Shenzhen, China) at a crosshead speed of 50 mm/min, according to GB/T1040.3-2006-B1standard. The films were cut into a dumbbell-shape at room temperature. The sample thicknesses were measured using a digital external micrometer (accurate to 0.001 mm), and the measurements were in triplicate, and the averaged value was reported.

### 2.10. Measurement of Oxygen Transmission Rate

The oxygen permeability values of the Si/PC copolymer membrane were measured using an oxygen permeability tester (VAC-VBS, Labthink Ltd., Jinan, China) according to the GB/T 1038-2000 standard with a test gas pressure of 1.01 × 105 Pa and upper and lower degassing times of 4 h. All the membrane samples were cut into circles with diameters of 5.5 cm. The sample thicknesses were measured using a digital external micrometer (accurate to 0.001 mm), and the measurements were in triplicate, and the averaged value was reported.

### 2.11. Measurement of Contact Angle

The contact angle was tested using an automatic contact angle meter (Theta, Biolin Scientific Ltd., Espoo, Finland) with pure water as a probe liquid at room temperature. The higher contact angle indicates the surface of the composite film has a better hydrophobicity. The measurements were carried out in triplicate and average values calculated.

## 3. Results and Discussions

### 3.1. ^1^H NMR Analysis

**Figure 2:**
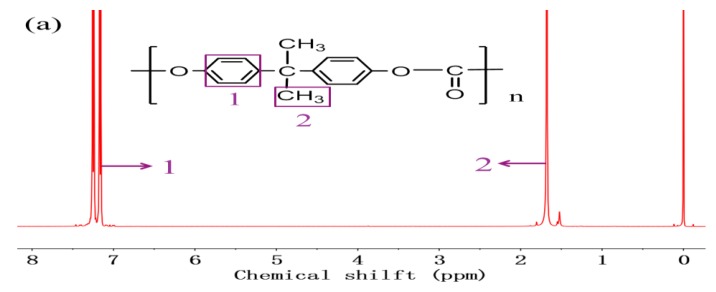

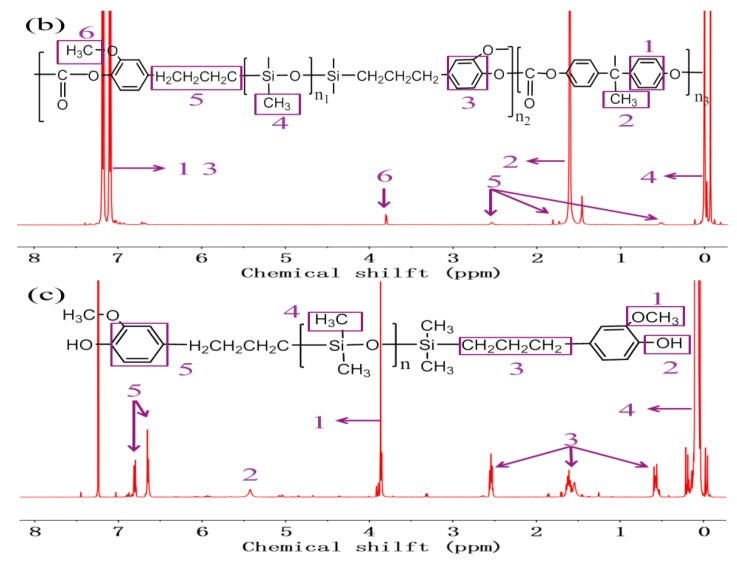
^1^H NMR spectrograms of 0%Si/PC (**a**), 20% Si/PC (**b**), and bio-phenol polysiloxane (**c**).

Figure 2 shows the ^1^H NMR spectrograms of 0%Si/PC (a), 20%Si/PC (b) and bio-phenol polysiloxane (c). As Figure 2a shows, the signals at 7.25, 1.7, and 0 ppm correspond to benzene, –C–CH_3_ and –Si–CH_3_ groups, respectively. In Figure 2b, four new absorption peaks are shown. The signal at 3.8 ppm corresponds to –OCH_3,_ which is from bio-phenol polysiloxane. The signals at 2.5, 1.8, and 0.5 ppm correspond to –CH_2_CH_2_CH_2_– which is from bio-phenol polysiloxane. The signal at 0 ppm was enhanced because of the –Si–CH_3_ group of bio-phenol polysiloxane. In addition, in Figure 2c, the signal at 5.5 ppm corresponds to –OH, which is from bio-phenol polysiloxane, and this signal disappeared in Figure 1b. It indicates that the –OH was reacted completely and the bio-phenol polysiloxane was copolymerized with the PC monomer.

### 3.2. FTIR Analysis

**Figure 3:**
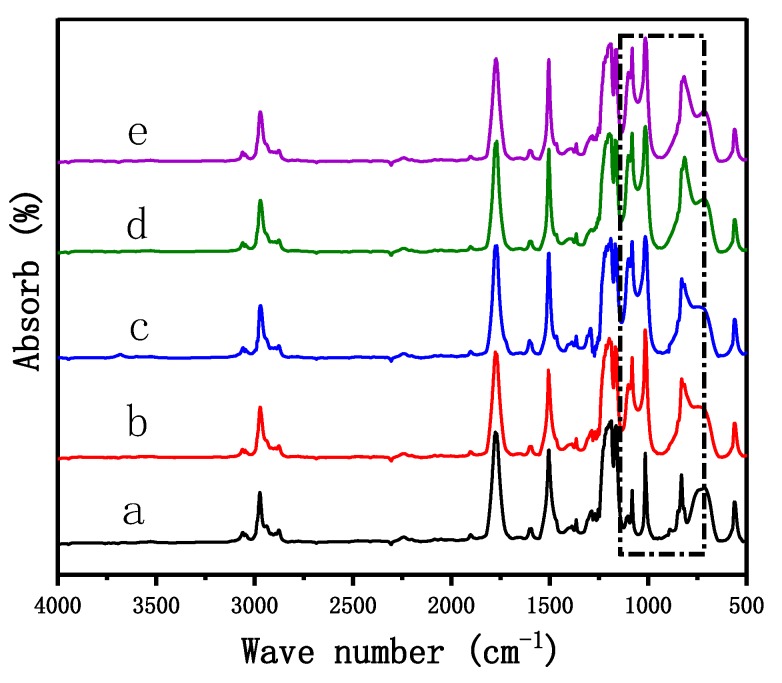
FTIR spectrograms of 0%Si/PC (**a**), 10%Si/PC (**b**), 14%Si/PC (**c**), 18%Si/PC (**d**), and 20%Si/PC (**e**).

For better proving the synthesis of the Si/PC copolymer, FTIR was employed to identify functional groups of productions. As Figure 3 shows, all five curves show similar absorption peaks. The absorption peaks appeared at 2900, 1775, 1520, and 1220 cm^−1^ corresponded to –CH_3_ bond stretching vibration adsorption, C=O bond stretching vibration adsorption, benzene ring C=C bond stretching vibration adsorption, and C–O bond stretching vibration adsorption, respectively. The difference in the five curves was mainly the absorption peaks in the black dashed box. Compared with curve a, the absorption peaks at the range of 1220 to 750 cm^−1^ were enhanced for the Si–O–Si bond stretching vibration adsorption and Si-C vibration adsorption, which derive from bio-phenol polysiloxane in curves b–e. Combined with the above ^1^H NMR analysis, it indicates that the bio-phenol polysiloxane molecular chain was copolymerized with the PC monomer, and the Si/PC copolymer was synthesized successfully.

### 3.3. GPC Analysis

In Table 1, the molecular weights and the distribution indexes of bio-phenol polysiloxane, 0%Si/PC, 14%Si/PC, and 20%Si/PC are reported. As the data show, the molecular weight of bio-phenol polysiloxane is 1989, and its distribution index is 1.61. As the amount of bio-phenol polysiloxane was gradually increased, the more bisphenol A was replaced by bio-phenol polysiloxane in the PC molecular chain, and thus the molecular weight of the Si/PC copolymer also increased. The molecular weights of Si/PC copolymers were in the range of 10,000 to 19,000, and their distribution indexes were in the range of 1.58 to 1.61. The result illustrates that bio-phenol polysiloxane molecular chain was copolymerized with the polycarbonate monomer.

### 3.4. Morphology Analysis

**Figure 4:**
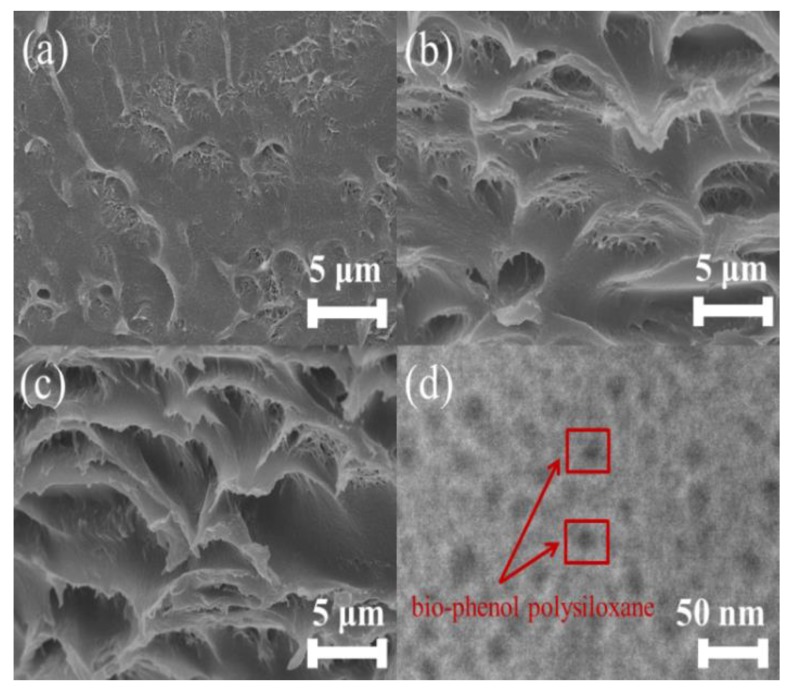
The fracture surface SEM images of 0%Si/PC (**a**), 14%Si/PC (**b**), and 20%Si/PC (**c**); the TEM image of 20%Si/PC (**d**).

Figure 4a–c report the fracture surface SEM images of 0%Si/PC (a), 14%Si/PC (b), and 20%Si/PC (c). Figure 4d reports the TEM image of 20%Si/PC. As shown in the SEM images, the fracture surface of 0%Si/PC was relatively smooth. As the amount of bio-phenol polysiloxane gradually increased, the fracture surface roughness of Si/PC also increased. It illustrates that the addition of bio-phenol polysiloxane had enhanced the flexibility of the Si/PC copolymer. In Figure 4d, we can observe that the black points are the dispersion phase, and the rest area is a continuous phase. It shows that the bio-phenol polysiloxane took the shape of an island in the continuous phase.

### 3.5. Thermal Analysis

**Figure 5:**
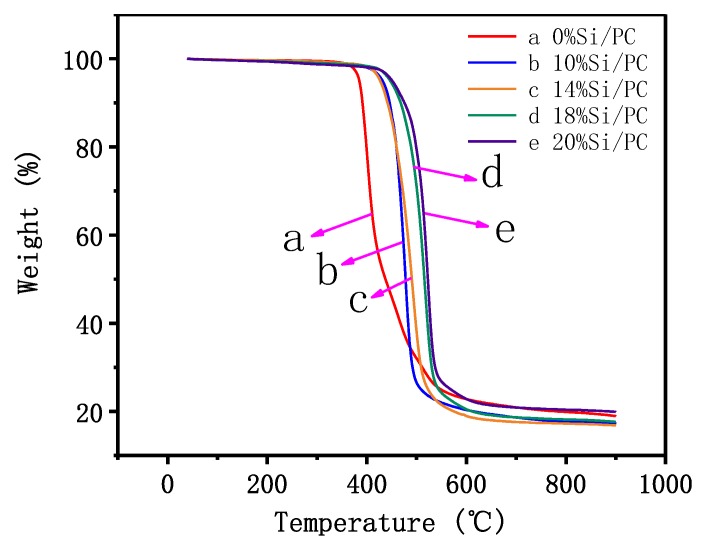
Thermal weight loss curves of 0%Si/PC (**a**), 10%Si/PC (**b**), 14%Si/PC (**c**), 18%Si/PC (**d**), and 20%Si/PC (**e**).

Figure 5 reports the TG degradation curves of 0%Si/PC (a), 10%Si/PC (b), 14%Si/PC (c), 18%Si/PC (d), and 20%Si/PC (e). In curve a, when the ratio of weight loss reached 5%, the pyrolysis temperature of 0%Si/PC was 384.7 °C. As the amount of bio-phenol polysiloxane gradually increased, the temperature of the Si/PC copolymer at the thermal weight loss of 5% also increased. When the amount of bio-phenol polysiloxane reached 20%, the temperature of the Si/PC copolymer at the thermal weight loss of 5% was 450.8 °C which was 76.1 °C higher than 0%Si/PC. It indicates that the modification with bio-phenol polysiloxane improved the thermal stability of the Si/PC copolymer. This is because the main chain of bio-phenol polysiloxane is –Si–O–Si– whose bond energy reaches to 1014.2 KJ/mol, and the polysiloxane possesses excellent heat resistance. In addition, the introduction of benzene, which derives from bio-phenol polysiloxane, also enhanced the thermal stability of the Si/PC copolymer to some extent [19].

### 3.6. Mechanical Properties

**Figure 6:**
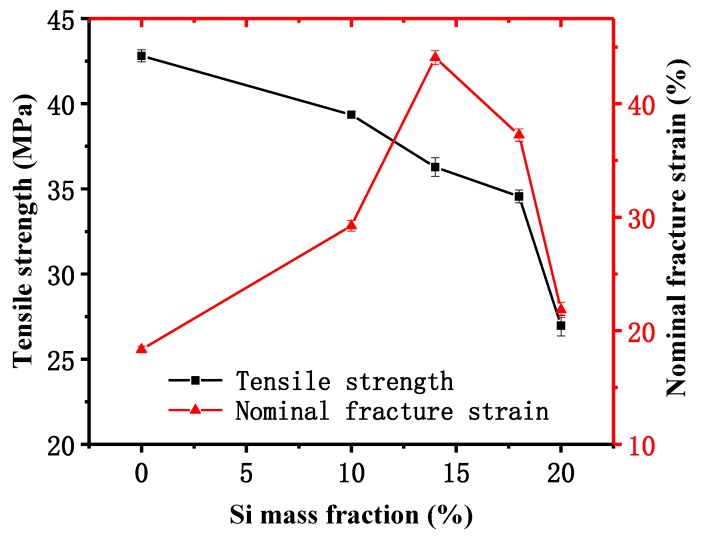
Mechanical properties of Bio-phenol polysiloxane/polycarbonate (Si/PC) copolymer with different Si mass fraction.

The mechanical properties of 0%Si/PC, 10%Si/PC, 14%Si/PC, 18%Si/PC, and 20%Si/PC copolymer membrane are shown in Figure 6. The tensile strength of 0%Si/PC membrane was 42.81 MPa. As the amount of bio-phenol polysiloxane increased gradually, the tensile strength of the Si/PC copolymer membrane decreased. When the amount of bio-phenol polysiloxane reached 20%, the tensile strength of the Si/PC copolymer membrane decreased by 37%, ranging from 42.81 to 26.97 MPa. The nominal fracture strain of 0%Si/PC membrane was 18.35%. When the amount of bio-phenol polysiloxane reached 14%, the nominal fracture strain of the Si/PC copolymer was 44.05%, which was increased by 140%. When the amount of bio-phenol polysiloxane was over 14%, the nominal fracture strain of the Si/PC copolymer membrane gradually decreased. When the amount of bio-phenol polysiloxane reached 20%, the nominal fracture strain of the Si/PC copolymer decreased to 21.85%. The result shows that the addition of bio-phenol polysiloxane reduces the tensile strength and improves the nominal fracture strain of the Si/PC copolymer membrane in a certain dosage range. It can be attributed to the excellent flexibility of the chain segment of bio-phenol polysiloxane.

### 3.7. Oxygen Transmission Rate

The oxygen transmission rate of 0%Si/PC, 10%Si/PC, 14%Si/PC, 18%Si/PC, and 20%Si/PC copolymer membrane are shown in Table 2. The oxygen transmission rate of 0%Si/PC was 220.0515 cm^3^/m^2^·24h·0.1Mpa, while for the 10%Si/PC, it was 204.383 cm^3^/m^2^ 24h 0.1MPa. The oxygen permeability of 10%Si/PC has been reduced for the homodisperse of a small amount of bio-phenol polysiloxane in the PC matrix and thus enhances the compactness of the Si/PC copolymer membrane. When the amount of bio-phenol polysiloxane was in the range of 10% to 20%, the oxygen permeability of the Si/PC copolymer membrane increased gradually when increasing the additive amount of bio-phenol polysiloxane. When this amount reached 20%, the oxygen transmission rate of the Si/PC copolymer membrane was 502.65 cm^3^/m^2^·24h·0.1MPa, which was increased by 128.4% compared with pure PC membrane. The oxygen permeability of Si/PC has been improved for the high permeability of bio-phenol polysiloxane. The result shows that the addition of bio-phenol polysiloxane could improve the oxygen permeability of the PC membrane to meet its application in special fields.

### 3.8. Contact Angle

**Figure 7:**
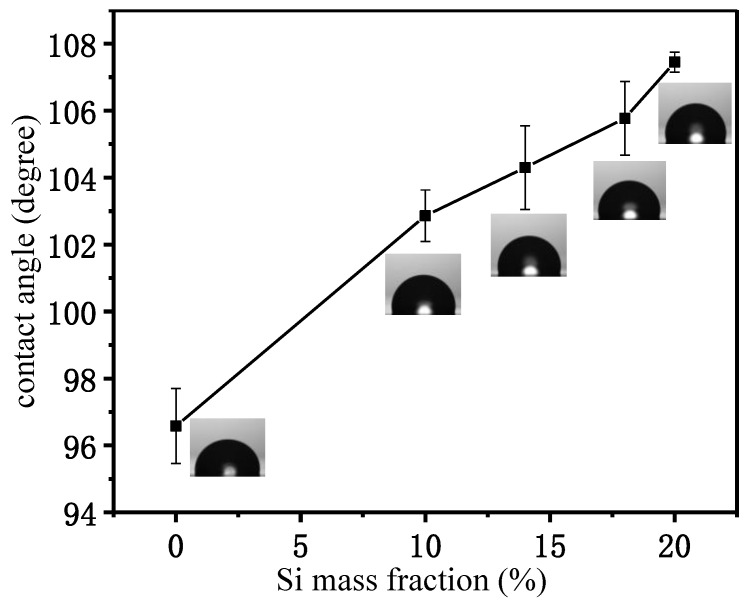
Water contact angle of the Si/PC copolymer with different Si mass fraction.

The water contact angle on the surface of the Si/PC copolymer membrane was measured, and the results are shown in Figure 7. The higher contact angle indicates the surface of the Si/PC copolymer membrane has better hydrophobicity. As Figure 7 shows, with increasing contents of bio-phenol polysiloxane, the water contact angle on the surface of the Si/PC copolymer membrane was increased gradually, ranging from 96.58° to 107.45°. Polysiloxane has excellent hydrophobicity, which is used as an auxiliary agent to improve the hydrophobicity of material in industrial production. The result indicates that the addition of bio-phenol polysiloxane has enhanced the hydrophobicity of the Si/PC copolymer.

## 4. Conclusions

Synthesized via the facile heterogeneous catalytic route, bio-phenol polysiloxane was designed to copolymerize with the PC monomer, and the Si/PC copolymer was synthesized successfully. It was shown that the bio-phenol polysiloxane took the shape of an island in the continuous phase. With increasing the additive amount of bio-phenol polysiloxane, the thermal stability, oxygen permeability, and hydrophobicity of the Si/PC copolymer improved. When the additive amount of bio-phenol polysiloxane reached 20%, the temperature of the Si/PC copolymer at the thermal weight loss of 5% was 450.8 °C which was 76.1 °C higher than 0%Si/PC. The oxygen transmission rate of 20%Si/PC copolymer membrane was 502.65 cm^3^/m^2^·24h·0.1MPa, which was increased by 128.4% compared with pure PC membrane. Through copolymerization modification with bio-phenol polysiloxane, the Si/PC copolymer exhibits excellent comprehensive performance, which is expected to apply in high-end fields, such as aviation, military, and the electronic field.

## Figures and Tables

**Table 1:** Molecular weight and its distribution index of bio-phenol polysiloxane/polycarbonate (Si/PC) copolymer.

Samples	*M*_n_	*M*_w_	*M*_z_	*M*_z_/*M*_w_
Bio-phenol siloxane	1989	3332	5468	1.61
0%Si/PC,	10,429	22,194	35,204	1.58
14%Si/PC	16,804	32,989	52,088	1.58
20%Si/PC	18,818	34,537	55,667	1.6

**Table 2:** Oxygen transmission rate of the Si/PC copolymer with different Si mass fraction.

Si Mass Fraction	Oxygen Permeability (cm^3^/m^2^·24h·0.1MPa)
0%	220.0515 ± 6.64
10%	204.383 ± 2.58
14%	344.378 ± 0.25
18%	464.051 ± 2.31
20%	502.65 ± 0.78
